# The synergy between the PscC subunits for electron transfer to the P_840_ special pair in *Chlorobaculum tepidum*

**DOI:** 10.1007/s11120-024-01093-7

**Published:** 2024-04-16

**Authors:** Alexandros Lyratzakis, Vangelis Daskalakis, Hao Xie, Georgios Tsiotis

**Affiliations:** 1 Department of Chemistry, School of Science and Engineering, University of Crete, Heraklion, 70013 Greece; 2 Department of Chemical Engineering, School of Engineering, University of Patras, Rion, Patras, 26504 Greece; 3 Max Planck Institute of Biophysics, 60438 Frankfurt am Main, Germany

**Keywords:** Photosynthesis, Green sulfur bacteria, Type I Reaction center, Molecular dynamics, Electron transpor

## Abstract

The primary photochemical reaction of photosynthesis in green sulfur bacteria occurs in the homodimer PscA core proteins by a special chlorophyll pair. The light induced excited state of the special pair producing P_840_^+^ is rapidly reduced by electron transfer from one of the two PscC subunits. Molecular dynamics (MD) simulations are combined with bioinformatic tools herein to provide structural and dynamic insight into the complex between the two PscA core proteins and the two PscC subunits. The microscopic dynamic model involves extensive sampling at atomic resolution and at a cumulative time-scale of 22µs and reveals well defined protein–protein interactions. The membrane complex is composed of the two PscA and the two PscC subunits and macroscopic connections are revealed within a putative electron transfer pathway from the PscC subunit to the special pair P_840_ located within the PscA subunits. Our results provide a structural basis for understanding the electron transport to the homodimer RC of the green sulfur bacteria. The MD based approach can provide the basis to further probe the PscA-PscC complex dynamics and observe electron transfer therein at the quantum level. Furthermore, the transmembrane helices of the different PscC subunits exert distinct dynamics in the complex.

**Supplementary Information:**

The online version contains supplementary material available at 10.1007/s11120-024-01093-7.

## Introduction

Photosynthetic reaction centers (RCs) are at the heart of photosynthesis. Antenna proteins harvest light energy which is converted into chemical energy in the RCs; a process that ultimately sustains most life on Earth. During evolution, photosynthetic organisms developed different RCs to adapt to various environments which are classified based on their terminal electron acceptors as type I (Fe-S type) or type II (quinone type) (Gisriel et al. 2021).

*Chlorobaculum tepidum* (*Cba tepidum*) is a thermophilic anaerobic green sulfur bacterium that utilizes reduced sulfur compounds as electron donors to the photosynthetic electron transport chain during photosynthesis (Wahlund et al. 1991). The photosynthetic apparatus of *Cba tepidum*, a type I RC, consists of four membrane subunits (the two 82-kDa PscA core subunits and the two 23-kDa PscC cytochrome *c*z proteins) and two soluble proteins (one 24-kDa PscB iron/sulfur protein and the 17-kDa PscD proteins) (Rémigy et al. 1999, 2002; Hauska et al. 2001). The two PscA subunits coordinate the electron transport chain cofactors while the two PscC mediate the transfer of electrons from the menaquinol/ cytochrome c oxidoreductase to P_840 special_ pair. The assembly further interacts with an intermediate light-harvesting complex, the Fenna–Matthews–Olson (FMO) protein. FMO is a water-soluble BChl *a*-binding protein that is located on the periphery, outside the cytoplasmic membrane (Hauska et al. 2001). FMO serves as an interconnecting node, mediating the transfer of excitation energy from the chlorosome to the RC. Due to the homodimeric nature of its photosynthetic apparatus, it is believed that *Cba tepidum* photosynthetic machinery is closely related to the ancestral RC and, thus, may provide insights into the evolution of photosynthesis (Eisen et al. 2002).

Protein–protein interactions (PPIs) play an important role in numerous biological processes and especially in the electron transport process for photosynthesis. The identification of a complete PPI map requires knowledge of the complex conformational network in protein–protein or protein–cofactor pairs. Photosynthesis is dependent on the formation of protein complexes which contain both membrane-integral and soluble protein subunits. Until recently, a detailed understanding of the mechanisms underlying the electron transport from the two PscC subunits to the P_840_ special pair has been hindered by the fact that no structural information was available. Although high resolution structures of the whole photosynthetic apparatus of the *Cba tepidum* are available, no PPI were observed therein for the heme-containing soluble PscC subunit, due to its high flexibility and unresolved structure (Puskar et al. 2022; Chen et al. 2023; Xie et al. 2023). Until today the only information about the PPI of the heme-containing soluble region of the PscC subunit and the RC core subunit is based on protein–protein docking analysis (Xie et al. 2023), where dynamical information is however not provided. According to the binding mode, determined by the rigid protein – protein docking, only one cyt c_z_ of the PscC can interact with the special pair. This raises the question how the symmetrical arrangement of the PscC subunits support the electron transport from the menaquinol/ cytochrome c oxidoreductase and cyt *c*-554 which accepts electrons from thiosulfate (S_2_O_3_^2−^) oxidation to photo-oxidazied P_840_^+^ special pair.

Here, we present a combined deep learning – Molecular Dynamice (MD) study that allows us to obtain detailed atomistic insights into the PPI dynamics that are associated with the membrane core complex of *Cba tepidum.* In order to study the dynamics of the RC core complex, the initial models were built based on the Cryo-EM structure which contains two copies of the PscA and two copies of the membrane integral part of the PscC subunits. The soluble PscC part was reconstructed employing the machine deep learning algorithm AlphaFold2 (Jumper et al. 2021; Akdel et al. 2022), further refined by enhanced sampling and classical MD.

## Materials and methods

The initial coordinates to build our models were taken from the Cryo-EM structure of the whole photosynthetic reaction centre apparatus from the green sulphur bacterium *Cba tepidum* (chains A and C, pdb: 7z6q) (Xie et al. 2023), and the x-ray structure of the electron carrier water soluble domain cytochrome c (chain A, residues 129–206), resolved from the green photosynthetic bacterium *Cba tepidum* (pdb: 3a9f) (Hirano et al. 2010). A symmetrical PscA system was initially considered as also resolved by CryoEM (Xie et al. 2023). The two PscC subunits were placed based on a prediction from AlphaFold (Jumper et al. 2021; Akdel et al. 2022) and asymmetry was introduced due to the initial minimization to avoid clashes between atoms (< 0.12 nm) or helices. Thus, only one heme containing soluble PscC subunit (termed ‘docked PscC’) was constructed close to PscA, the other soluble PscC domain moved further away due to clashes in an asymmetrical re-construction. For more details, please refer to the Supplementary Material 1. The system was simulated at neutral pH with key protonations to produce the correct hydrogen bonding network within the polypeptide chains so that no large deviations are observed from the CryoEM, or x-ray experimentally resolved structures. The PscA-PscC super-complex was embedded in a POPC (1-palmitoyl-2-oleoyl-glycero-3-phosphocholine) lipid membrane patch and hydrated. The models contained 150 mM KCl, with a surplus of Cl^−^ ions (13.6mM) to neutralize the system. Two models were considered that contained between ∼ 344k (2xPscA-1xPscC) and ∼ 347k (2xPscA-2xPscC) atoms. The equilibrated unit cell dimensions of each model were roughly 15.5 × 15.5 × 14.4 nm^3^. The Charmm36 Force Field (Lindahl et al. 2010) was employed for the polypeptide chains and heme groups. The bacteriochlorophylls pigments were parameterized based on the literature (Chandrasekaran et al. 2015), thylakoid lipids bound within the protein scaffold and POPC lipids of the membrane patch were described by Charmm compatible parameters in the Charmm gui (Jo et al. 2008). The systems were equilibrated (see Supplemental Material for details) at 319 K and 1 atm. Sampling of PscA-PscC conformations was performed by classical and enhanced sampling (replica exchange with solute tempering, REST2) methods (Wang et al. 2011). Conformations were sampled for the two different models (PSII-2xPscC, PSII-1xPscC): 2 models x 1µs Classical Molecular Dynamics (MD) equilibration + 2 models x 20 REST2 enhanced sampling replicas x 200ns + 2 models x 3 representative conformations x 2 independent trajectories x 1 µs Classical MD production = 22 µs. The first 200ns from each classical trajectory and the first 50ns from each REST2 replica were disregarded as further equilibration and have not been used in the analysis. Thus, the analyzed simulation time is 2 × 20 × 150ns = 6µs from REST2 replicas and 12 × 0.8µs = 9.6 µs from classical MD (4.8µs per each PscA-2xPscC, PscA-1xPscC model).

All replicas diffuse over the 319-500 K temperature range, indicating an efficient exploration (See Supplementary Materials). Given the large size of the models and the immense computational resources needed to sample at the REST2 level, we believe that we have pushed the methodology to the limit and obtained adequate sampling for the extraction of key conformations regarding the correlation of the PscC-PscC and PscA-PscC domain motions. Furtheremore, we note that this approach produces a rather symmetric projection of sampled points over two key vectors – the PscC-PscC and PscA-PscC distances – without seemingly any important un-explored areas (see Fig. 2 below and related discussion).

## Results and discussion

The photosynthetic RC is an essential component of biological organisms involved in the process of photosynthesis, serving to convert solar energy into stored chemical energy. The membrane core of *Cba tepidum* RC consists of two PscA and two PscC subunits (Xie et al. 2023). The 22 transmembrane helices (TMH) of the two PscA subunits can be divided into an antenna domain comprising the first six TMHs and an electron transfer (ET) domain comprising the last five TMHs (Xie et al. 2023). The three TMHs of each PscC subunit are symmetrically located around the PscA homodimer and unlike in other RCs, only the RC of the green sulfur bacteria employs a transmembrane protein subunit associated with the delivery of electrons (Gisriel et al. 2021).

**Fig. 1:**
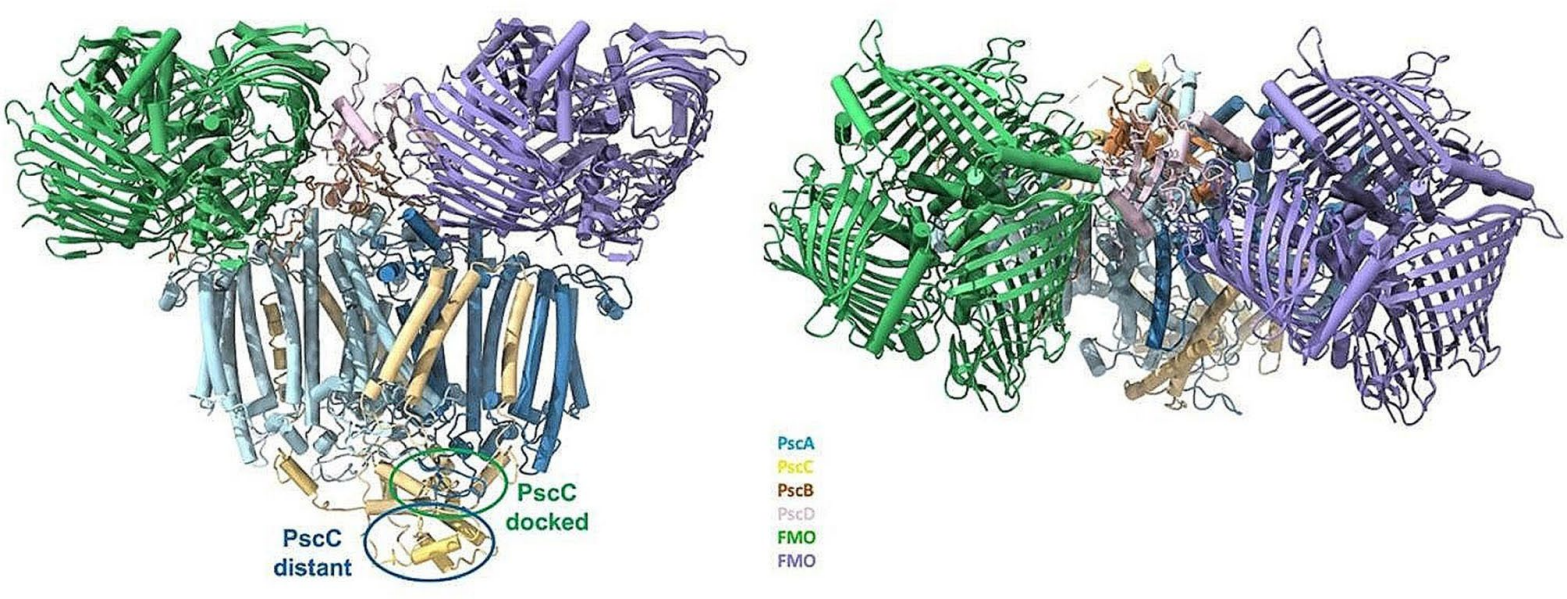
Strucuture of the photosynthetic apparatous of *Cba tepidum* with the PscC subunits containing the soluble domain (Xie et al. 2023). Side and top view of the “whole” complex

Simulations have shown that the PscA homodimer is similar to the cyanobacterial PSI and the purple bacterial RC, which both have only one binding site for cyt. c_z_ (Axelrod et al. 2002; Kölsch et al. 2018; Xie et al. 2023). For this reason, we have initially placed only one heme containing soluble PscC subunit (termed ‘docked PscC’) close to PscA and the P_840_ special pair, while the second PscC soluble heme containing domain (distant PscC) is placed further away (Fig. 1). Figure 1 shows that the addition of the soluble heme containing domain in the complex structure has not affected in any way the initial orientations of the membrane integral part that matches the reported resolved structure (Xie et al. 2023). The all-atom re-constructed model of a complete PscA-PscC complex embedded within a thylakoid lipid bilayer mimetic contains either two or one PscC subunits. In MD simulations, we can consider the confined nature of the lipid bilayer and water, thus PPI therein can be probed in relatively smaller time-scales, compared to the experiments. Enhanced sampling MD approaches, as described in the methods section, achieve large speed-ups and an efficient sampling of PPI.

**Fig. 2:**
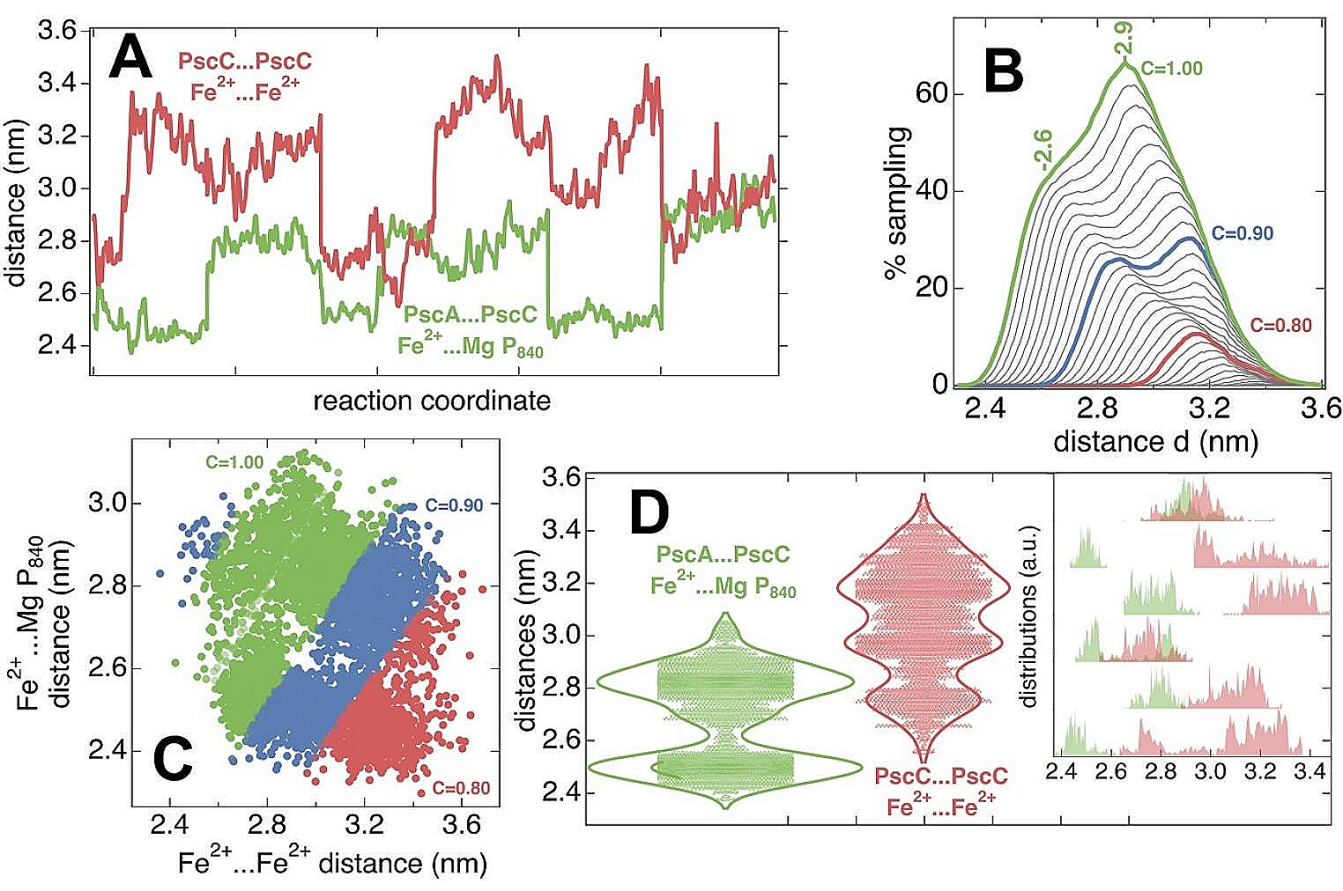
(**A**) The co-evolution of Fe^2+…^ Fe^2+^ and Fe^2+…^Mg P_840_ distances along an arbitrary reaction coordinate (6 concatenated simulation classical MD trajectories). (**B**) The percentage of time the PscC-PscC (Fe^2+…^ Fe^2+^) and PscA-PscC (Fe^2+…^Mg P_840_) subunits sample conformations with iteratively (and mutually exclusive) small (< C*d) and large (> d) distances. Each curve represents a different C parameter value between 0,67 to 1. Between successive curves C differs by 0.01. Selected curves are colored red for C = 0.80, blue for C = 0.90 and green for C = 1.00 (**C**) The sampled frames of all classical MD trajectories projected over two vectors; the Fe^2+…^ Fe^2+^ and Fe^2+…^Mg P_840_ distances. Each circle corresponds to one frame. The coloring scheme is identical to panel B and colored circles correspond to the respective C values selected (red for C = 0.80, blue for 0.90 and green for 1.00). (**D**) Violin plots of the Fe^2+…^ Fe^2+^ (red) and Fe^2+…^Mg P_840_ (green) distances

The enhanced sampling MD simulations thus allowed extensive sampling of the PscA-PscC complex and the analysis produced three different conformations (see Supplementary Materials). The three different conformational states of the PscA-PscC complex were mainly populated over the REST2 time considered. These states can be considered as distinct ensemble average conformations originated from the plethora of different copies of the system in different conformations during the REST2 sampling. Initiated from each of these three states, we produced two independent trajectories of 4.8 µs cumulative time-sampling for the PscA-2xPscC model. Please note that independent short re-equilibrations of the three conformations (re-asignment of velocities, temperature increase from 100 to 319 K over a period of 10 ns) was also performed prior to each 1 µs production sampling. Thus, the actual initial structures are different for all (six) independent runs. 200ns were also considered further equilibration from each 1 µs trajectory and were disregarded. The dynamics within these trajectories show the RC complex to exert a distinct pattern of spatial distributions of the PscA and the two PscC subunits or domains, in terms of the respective distances between the heme ring of the PscC subunits (Fe^2+^) and the central Mg coordinating the BChl*α΄* of the P_840_ of the special pair (Fig. 2A). Figure 2A shows all sampled co-evolution of these two important distances from six independent trajectories over an arbitrary reaction coordinate, which corresponds to the concatenated simulation time of the six trajectories. The cumulative simulation time is 6 × 0.8 µs = 4.8 µs, as the first 0.2 µs have been disregarded from each trajectory. Please note that this cannot be considered as a course of events from start to finish, but rather as a qualitative accumulation of uncorrelated in time sampled pairs of distances in windows of 0.8µs. However, this does not mean that we cannot perform statistics on the time-series, but instead these time-series cumulatively provide the configurational space of the PscA-PscC super-complex. This is a common practice in MD methods, for the adequate sampling of the configurational space of large biomolecules. For the dynamics of individual trajectories (0–1µs) and the Root Mean Square Deviation (RMSD) of the protein backbone, please refer to the Supplementary Material.

The relative PscA-PscC (Fe^2+ …^ P_840_ Mg) and PscC-PscC (Fe^2+ …^ Fe^2+^) distance time – series seem to be correlated to a large extend, as seen in Fig. 2B. For the Fig. 2B, we have calculated the percentage of simulation time or sampled frames (over the 4.8µs cumulative time of classical MD) where the PscC-PscC distance is either lower than a value C*d, or larger than d, and at the same time the PscA-PscC distance is respectively either larger than d, or lower than a value C*d. These percentages are translated into curves versus the distance d and factor C. Peaks in the curves refer to the cases where PscC subunits get closer together and the docked PscC moves away from the special pair, or the docked PscC moves closer to the special pair and away from the second PscC subunit. C is a factor between 0.67 and 1, with selected curves in Fig. 2B to reflect different C values using a 0.01 step for two successive curves. We selectively use coloring for curves that correspond to values of C = 0.8, 0.9 and 1. The interpretation of the curves is present in Table1 with the same coloring as in Fig. 2B. In detail, for C = 1, with peaks at d = 2.9 and d = 2.6 (i) more than 60% of the simulation time we sample configurations where the Fe^…^Fe distance is lower than 1*2.9 nm or greather the 2.9 nm and at the same time the Fe^…^Mg P_840_ distance is greater than 2.9 nm, or less than 1*2.9 nm respectively, (ii) more than 40% of the simulation time we sample configurations where the Fe^…^Fe distance is lower than 1*2.6 nm or greater the 2.6 and at the same time the Fe^…^Mg P_840_ distance is greater than 1*2.6 nm, or less than 1*2.6 nm respectively. In the case of C = 0.90 for example, with a peak at d = 2.8 nm, more than a quarter (25%) of the total simulation time we sample configurations where the Fe^…^Fe distance is lower than 0.90*2.8 nm (= 2.5 nm) or greater the 2.8 nm and at the same time the Fe^…^Mg P_840_ distance is greater than 2.8 nm, or less than 0.90*2.6 nm (= 2.5 nm) respectively. We sample the PscA-2xPscC subunits at a large percentage of the time between conformations of alternating (and mutually exclusive) large and small PscA-PscC and PscC-PscC distances (see also Fig. 2A). We must make clear that this behavior represents a considerable amount of sampling time but not all of the simulation time. For the rest of the simulation time the PscA-PscC and PscC-PscC distances can be un-correlated in the sense that PscCs sample short or longer distances between them, or to the special pair at the same time. Figure 2C shows a cumulative projection of the sampled conformations in the six independent trajectories of 4.8µs over the two key distance vectors. Red circles (frames) at the lower right space correspond to the red curve of Fig. 2B, which means that they refer to frames where around 10% of the total simulation time the Fe^…^Fe distance is lower than 0.80*3.1 nm (= 2.48 nm) or greater than 3.1 nm and at the same time the Fe^…^Mg P_840_ distance is greater than 3.1 nm, or less than 0.80*3.1 nm (= 2.48 nm) respectively (see also Table1). Blue circles, that correspond to the blue curve of Fig. 2B, overlap the red-circles area and extend beyond towards the upper left space. Green circles practically cover the sampled space with overlaps in the red and blue-circle areas.

**Table 1:** The sampled distances between PscA/ PscC subunits associated with the percentage of simulation time. The coloring scheme is tha same as in Fig. 2B that corresponds to curves with C = 1.0 (green), 0.9 (blue) and 0.8 (red)

C Values	Peaks at d(nm)	Fe...Fe < C*d	Fe...BChl > d	Fe...Fe > d	Fe...BChl < C*d
**1.0**	**2.9**	**< 2.9 at 70%**	**> 2.9 at 70%**	**> 2.9 at 70%**	**< 2.9 at 70%**
**1.0**	**2.6**	**< 2.6 at 40%**	**> 2.6 at 40%**	**> 2.6 at 40%**	**< 2.6 at 40%**
*0.9*	*2.8*	*< 2.5 at 25%*	*> 2.8 at 25%*	*> 2.8 at 25%*	*< 2.5 at 25%*
*0.9*	*3.1*	*< 2.8 at 30%*	*> 3.1 at 30%*	*> 2.8 at 30%*	*< 3.1 at 30%*
***0.8***	***3.1***	***< 2.48 at 10%***	***> 3.1 at 10%***	***> 3.1 at 10%***	***< 2.48 at 10%***

An efficient configurational space sampling is based on the time integration of numerous independent trajectories of the same system that are either initiated at different configurations (three conformations), or refer to different initial distribution of velocities for the atoms of the same configuration (two independent trajectories). There seems to be adequate sampling of the configurational space, judged by the symmetrical distribution of sampled points in Fig. 2C. The absence of sampled configurations in the middle of the accumulation of points gives the trajectories a ‘donut’ shaped projection and periodicity. In Fig. 2D we show violin plots of the PscA-PscC (Fe^2+ …^ P_840_ Mg) and PscC-PscC (Fe^2+ …^ Fe^2+^) distaces over the cumulative sampling of 4.8µs. We can observe the distribution of the distances, with increased widths at values of statistical significance. This interprets to our sampling exerting two main values of the PscA-PscC (Fe^2+ …^ P_840_ Mg) distance at around 2.5 and at around 2.8 nm. For the PscC to PscC distance we sample three main values with statistical significance: at around 2.7, 2.9 and 3.2 nm. We assume that lower values enable an efficient electron transfer from PscC to the special pair (PscA), or to the second PscC, wheares no electron transfer from PscC to the special pair, nor to the second PscC is possible in case of the higher distances. A histogram for the associated distribution of distances in the 6 idependent trajectories is also provided for reference (inset of Fig. 2D).

In our MD simulations herein we observed that the soluble heme containing domain of the PscC subunits are asymmetrical and show distinct (and correlated) dynamics at a large percentage. The binding of the first heme containing PscC subunit (docked) prevents the binding of the second heme containing PscC subunit to P_840_ of the special pair due to steric hindrance. The method employed herein enables the docking and un-docking of subunits as it tempers the hydrophobic and electrostatic interactions between PscA and PscC-PscC subunits. However, no full un-docking of the one PscC and docking of the other subunit was ever sampled. We cannot exclude the possibility that at longer time scales, inaccessible by our setup, both PscC subunits could be un-docked from / and docked to PscA.

**Fig. 3:**
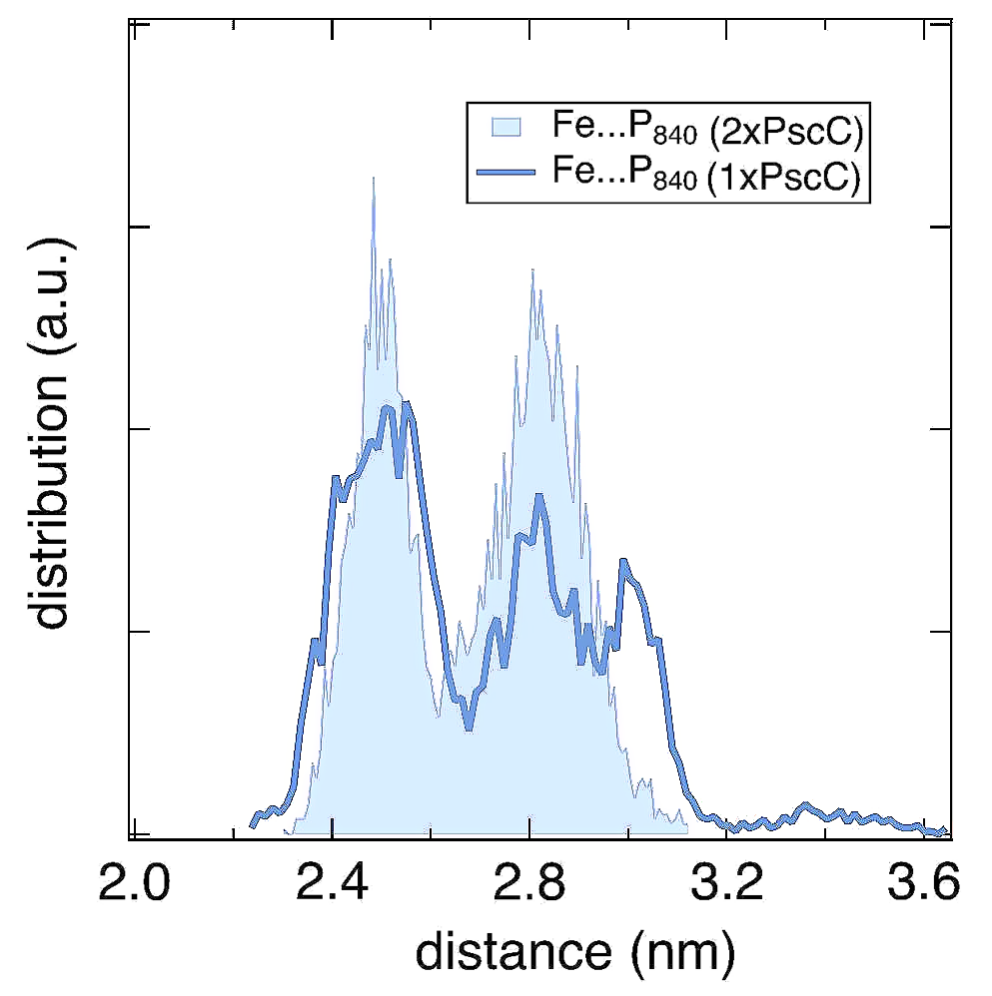
The probability density distribution of the Fe (PscC)… P_840_ (PscA) distances calculated in the cases of two or one PscC subunits in the model

Another question that comes forward is how does the docked PscC subunit behaves when the second (distant) PscC subunit is very far away? We simulate this case in the PscA-1xPscC model in the absence of the second PscC distant subunit. In the latter case, the docked PscC subunit samples conformations marginally closer but maybe important for an efficient electron transport (the rate of electron transfer is negatively correlated with the distance), and also larger distances, to the special pair Mg P_840_, when compared to the sampling in the presence of two PscC subunits (Fig. 3). The histograms are based on cumulative sampling of 9.6µs per model (4.8µs for PscA-1xPscC and 4.8µs for PscA-2xPscC). Thus, the presence of a second PscC subunit affects the dynamics of the first (docked) PscC subunit, adding to the correlated motion scenario. Please note that the absence of the second PscC subunit simulates the case where the second PscC subunit is further away from the docked PscC, with almost no interaction between them.

In contrast to the docking results which indicate that only one cytochrome has the possibility to transfer electrons to the special pair (Xie et al. 2023), in the recently reported structures of the same complex it was suggested that both cytochromes contribute in parallel to the electron transport pathway to the special pair (Puskar et al. 2022; Chen et al. 2023). Given the correlated (periodic) motion of the PscC subunits identified herein at large percentage, and no sampling of any docking exchanges to the PscA subunit, the MD results could assume an electron transport in series from the distant PscC heme to the docked PscC heme and then to P_840_; an arrangement different from a proposed model in which two identical heme groups donate electrons to P_840_ in parallel (Oh-oka et al. 1997; He et al. 2014; Puskar et al. 2022; Chen et al. 2023). By “electron transfer in series” we refer to the case where the distant reduced second PscC approaches the docked oxidized PscC, with the latter to move further away from the PscA special pair after the latter is reduced. An electron transfer occurs from the distant to the docked PscC subunit. Then, the distant oxidized PscC moves further away from the docked PscC, while the latter moves closer to the PscA to rapidly transfer its electron to the oxidized P_840_^+^ special pair. The suggested electron transport is in agreement with a recent study on the homodimer RC from the microaerophilic *Chloracidobacterium thermophilum* which includes two different cytochromes in series as electron donors to the P_840_ special pair (Dong et al. 2022). Further, it shows similarities to some purple bacterial RCs, in which c-type cytochromes serve as both electron donors and acceptors, and their heme groups are derived from a single peptide (Azai et al. 2010; Yu et al. 2018). The docked PscC soluble unit could cycle between conformations that are closer to the special pair and further away from the second (distant) PscC subunit, or closer to the second (distant) PscC subunit, but further away from the special pair (Fig. 2A-B). The distant PscC soluble subunit seems to follow a similar (but reverse) pattern sampling iteratively smaller or larger distances to the docked PscC for the correlated part of the motion sampled.

The distribution of the distances between the hemes and of the BChl*α΄* of the special pair varies throughout the sampling, as it is also true for the distance between the docked heme Fe^2+^ and the Mg of the special pair P_840_. Τhe results support a well-defined chorography of both cytochromes and PscA most of the time sampled. As already described, we have sampled cases in which when the two cytochromes are closer together the first cytochrome is far away from the reaction center. As it is true for the opposite scenario; when the first cytochrome is located closer to the reaction center, the cytochromes are far from each other. The trajectories cumulatively indicate that the subunits of the core complex exert a preferred correlated well defined PPI pathway.

For these correlated cases, when the PscC electron donors interact strongly with eachother they are not able to donate electrons to the reaction center. In contrast, when the docked PscC cytochrome is located close to the reaction center, the distant PscC cytochrome is further away from the first (Fig. 2A). This is an indication that when the reaction center is accepting electrons from the docked PscC, the distant PscC could be accessible to receive electrons from menaquinol: cyt c oxidoreductase or cyt c-554/555 (Tsukatani et al. 2008). This finding is in contrast to the proposed electron transfer pathway directly from the Rieske/cyt*b*-type complex via membrane-anchored cyt *c*-556 (CT0073) and from thiosulfate (S_2_O_3_^2−^) oxidation via cyt *c*-554 (CT0075) to one of the two monomeric PscC subunits (Kishimoto et al. 2023). Flash-induced absorption changes indicated that the photo-oxidized PscC can accept electrons from both the menaquinol: cyt c oxidoreductase and the cyt c-554/555 independently (Tsukatani et al. 2008). Both electron-transfer pathways which link the sulfur metabolisms to donate electrons to RC seems to act independently (Tsukatani et al. 2008; Azai et al. 2010). In the case that the PscC subunits act in series, the reduction of the distant PscC subunit via the membrane-anchored cyt *c*-556 and cyt *c*-554 would be a kinetic depended process (Oh-oka et al. 1997). As mentioned above the electron donors for the PscC subunits are the membrane-bound cytochrome c-556 and the water-soluble c-554 cytochrome. To be able to confirm which of the conformations we observed can receive electrons, docking experients with the known structure of both cytochromes were performed (Yu et al. 2013; Kishimoto et al. 2023). The docking calculation shows that only the distant cytochrome is accessible and only this cytochrome shows consistent protein interactions with both electron donors (Fig. S3).

**Fig. 4:**
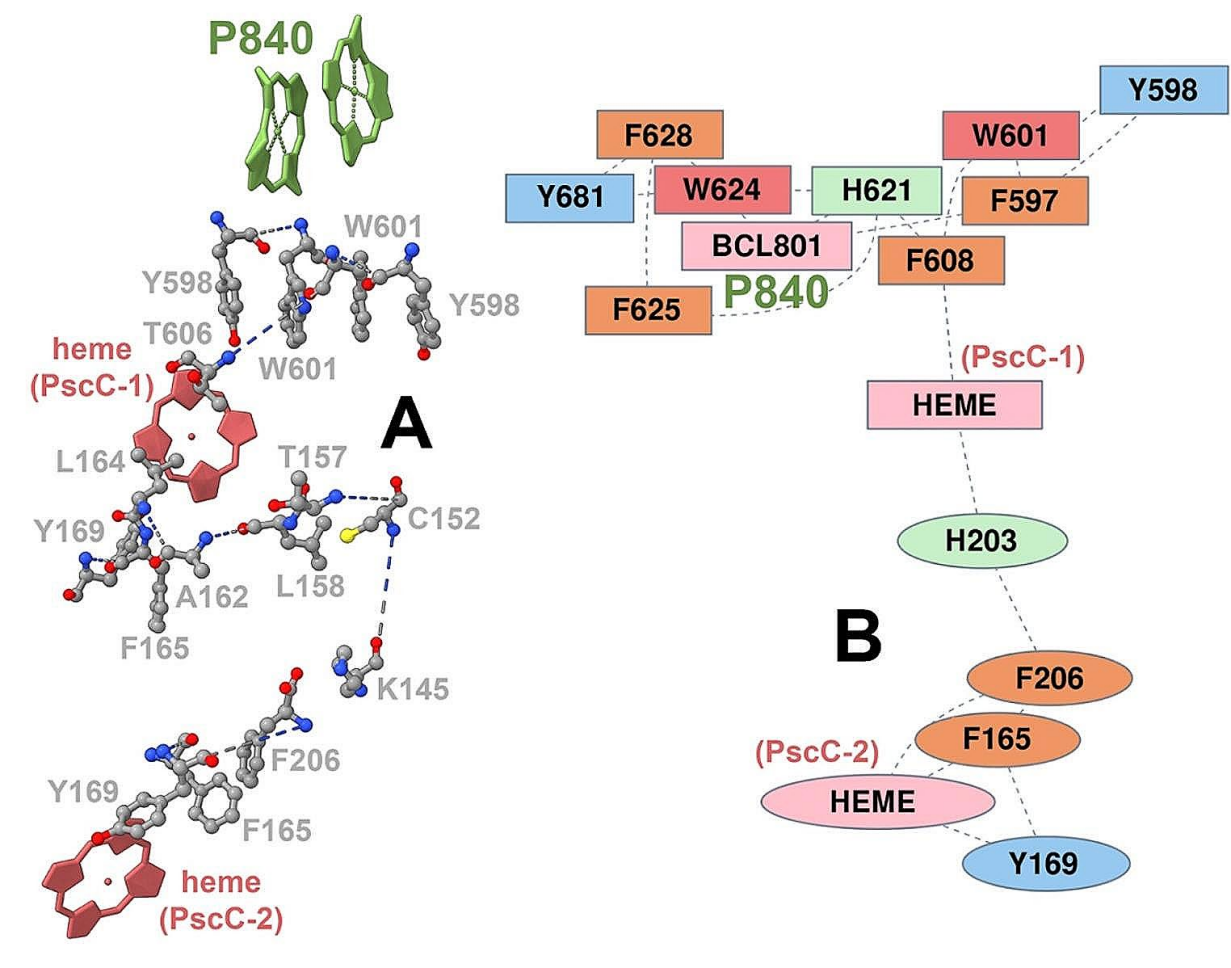
Proposed allosteric pathway (**A**) and electron transfer pathway (**B**) from the PscC subunits to the special pair. Different coloring has been used for the various aromatic residues and pigment. Two different clusters of electron transfer are indicated by oval and orthogonal shapes

A graph theoretical approach was employed to further probe the residue-residue correlations along the PscC-PscA model (for details please refer to the methods section in the Supplementary Materials). The residues along the shortest pathway from the distant PscC, to the docked PscC subunit and finally to the special pair P_840_ were determined as describe in ref (Bastian et al. 2009). This forms an allosteric pathway by a graph-theoretical approach (Negre et al. 2018). Perturbations at one residue can create long-range allosteric effects by their propagation through the network. The pathway includes residues like Trp (W), Tyr (Y) and Phe (F) in close interactions which could be associated with a putative electron pathway (Fig. 4A-B) calculated by the eMap method (Tazhigulov et al. 2019). Please note that the PscA-PscC conformation used to calculate the allosteric – electron pathways is an ensemble average – representative structure of the whole sampled configurational space of PscA-PscC and exerts shorter distances between PscA-PscC and PscC-PscC domains.

**Fig. 5:**
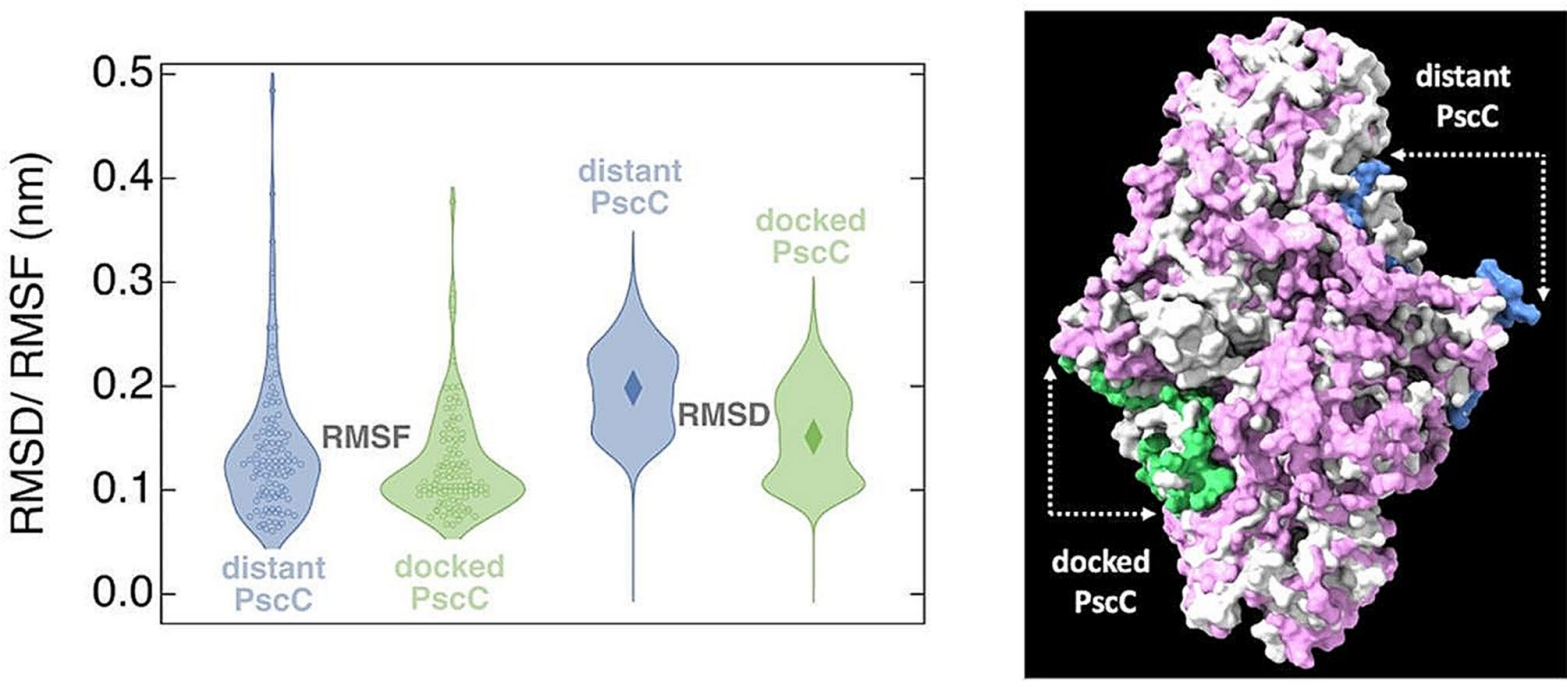
(Left panel). Violin plots for the RMSF and RMSD values calculated for the transmembrane helices (residues 1-100) of the docked PscC and the distant PscC subunits. The data points sampled are shown as small circles for the RMSF plots and the mean value is shown in diamond symbols for the RMSD plots. (Right panel). Surface of the PscA(pink)-PscC(blue/green) model complex superimposed to the PscA-PscC complex (gray) from (Xie et al. 2023). Arrows shows the difference between the distant and docked PscC subunit

The PscC subunit in the *Cba tepidum* RC contains three TMH which interact with the TMHs of both PscA subunits (Xie et al. 2023). In addition, the TMHs interact with pigments of PscA that are involved in the light harvesting and in the electron transfer (Xie et al. 2023). The transmembrane helices of the PscC subunits exert distinct dynamics between the docked PscC and the distant PscC subunit. The THMs associated with the docked PscC exert structural stability, whereas those of the distant PscC subunit exert increased mobility (flexibility), as judged by the violin plots of the values of RMSD (Root Mean Square Deviation) and RMSF (Root Mean Square Fluctuations) calculated for the respective backbones of THMs (Fig. 5 and Supplementary Material Movies S1 and S2). Violin plots can visualize the distribution of numerical data over a range, with increased widths at values of statistical importance. The violin shapes for the distant PscC covers a larger area towards higher RMSF/ RMSD values. The docked PscC-related shapes appear broader at lower RMSF/ RMSD values, indicating a restricted mobility – flexibility, compared to the distant-related violin shapes that exert broadening at higher values. This comes at odds with the two cytochrome subunits PscX and PscY from another species (*Chloracidobacterium thermophilum)*, where they seem to be membrane-tethered via a hydrophobic anchor from their N-terminus (Dong et al. 2022), thus exerting limited mobility.

## Conclusions

This work elucidates the features and PPI of the PscA-PscC complex that can formulate the basis for understanding the photosynthetic electron transfer pathway in green sulfur bacteria. A throughout insight is given into the atomic scale details of a periodic motion between PscA homodimer and the seemingly-symmetrically arrangement of the PscC subunits. In this way, a plausible picture of PscA-PscC organization and interaction is emerging for the first time at all-atom resolution.

**Fig. 6:**
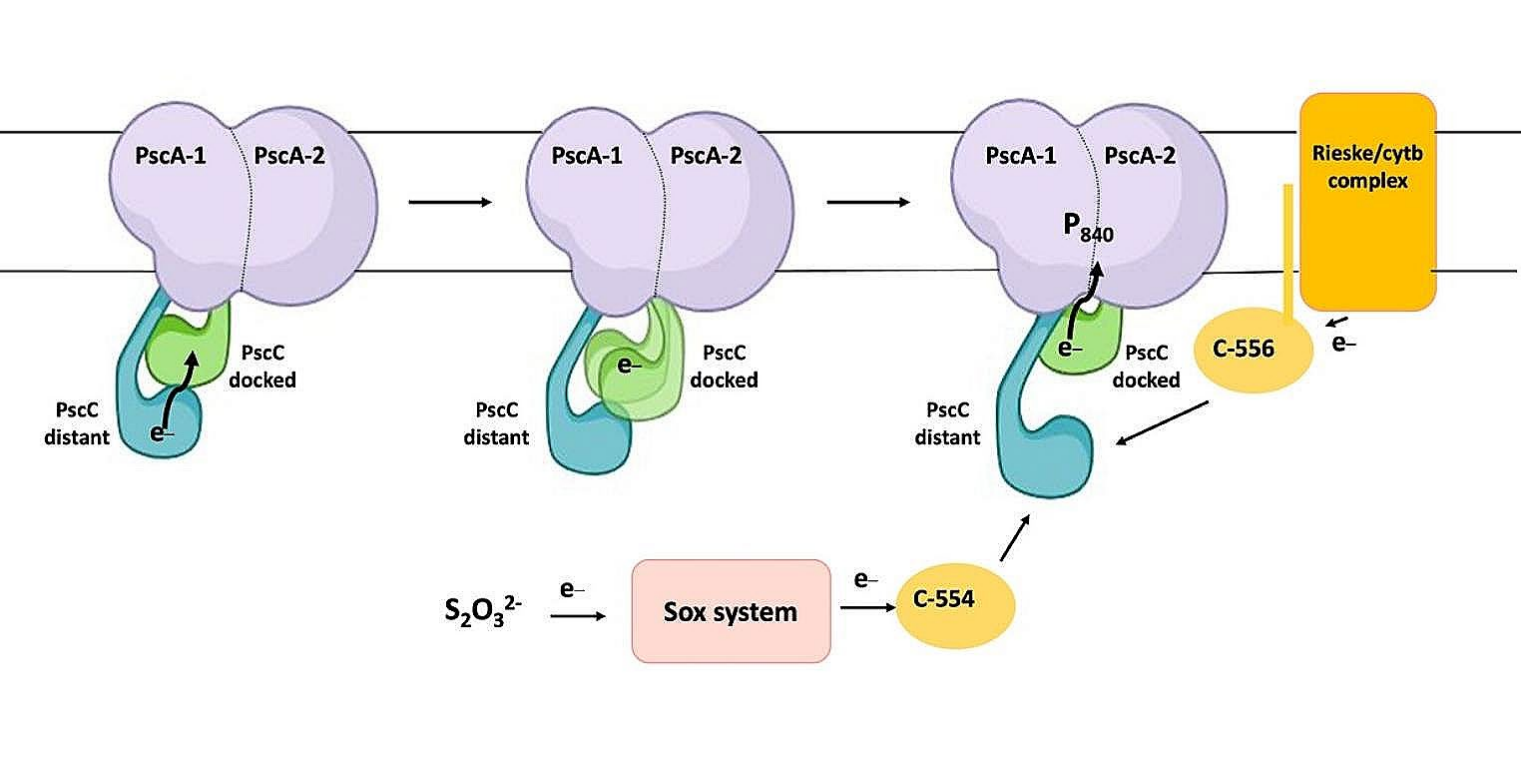
Model of the elctron flow from thiosulfate oxidation via c-554 and from from the menaquinol/cytochrome c oxidoreductase via c-556 to the P_840_ special pair

In contrast to the proposed model where the PscC subunits act in an independent manner in order to donate electrons to the P_840_ special pair, our results show a synergy between the PscC subunits in the electron transfer to the special pair (Fig. 6). The PscC subunits can act in a dependent manner creating two dynamically correlated but distinct and dissimilar subunits with one close to the RC and the other further away from it. The binding of one PscC monomer close to the special pair of the RC induces a dissociation of the other PscC subunit. Subsequently, when both PscC are coming closer together, a dissociation of the docked PscC subunit from the special pair is occurring most of the sampled time. This correlated motion of the PscC subunits is important for the donation of electrons to the special pair and to accept electrons from the Rieske/cyt*b*-type complex via membrane-anchored cyt *c*-556) and from thiosulfate oxidation via cyt *c*-554. Furthermore, the transmembrane helices of the PscC subunits exert distinct dynamics, with those of the docked PscC to exert stability, whereas those of the distant PscC subunit to exert increased mobility. This differentiation in flexibility could be associated with various effects on the electron transport pathway along PscA, however further simulations at the quantum level are needed to give this insight.

### Electronic supplementary material

Below is the link to the electronic supplementary material.

Supplementary Material 1

Supplementary Material 2

Supplementary Material 3

## Data Availability

Structures and related data from this study are available from the corresponding author upon reasonable requests.
