# Supplementary material for: The synergy between the PscC subunits for electron transfer to the P840 special pair in Chlorobaculum tepidum
Source: Photosynth Res. 2024 Apr 16;160(2-3):87–96. doi: 10.1007/s11120-024-01093-7 (PMC11108878; doi:10.1007/s11120-024-01093-7)
Supplement: Supplementary file 3 — Supplementary Material 3 [file 11120_2024_1093_MOESM3_ESM.docx]

**Supplementary Materials**

**The synergy between the PscC subunits for electron transfer to the P_840_ special pair in *Chlorobaculum tepidum***

Alexandros Lyratzakis^1, †^, Vangelis Daskalakis^2, †^, Hao Xie^3^ and Georgios Tsiotis^1, *^

**Materials and methods**

**Figure S1**

**Figure S2**

**Figure S3**

**Figure S4**

Movies S1 to S2

Supplementary References

**Materials and methods**

***Model coordinates.*** The initial coordinates to build our models were taken from the Cryo-EM structure of the whole photosynthetic reaction centre apparatus from the green sulphur bacterium *Cba tepidum* (chains A and C, pdb: 7z6q) (Xie et al. 2023), and the x-ray structure of the electron carrier water soluble domain cytochrome c (chain A, residues 129-206), resolved from the green photosynthetic bacterium *Chlorobium tepidum* (pdb: 3a9f) (Hirano et al. 2010). An alphafold2 prediction(Jumper et al. 2021; Akdel et al. 2022) was employed for the uniport sequence O07091 of 1-206 residues (cytochrome c, PscC) and the best structure was chosen (AF-O07091-F1). The AF-O07091-F1 structural prediction contains both the soluble and the transmembrane domains of cytochrome c and it was used as a guide. The soluble part from pdb 3a9f and the PscC transmembrane part from pdb 7z6q (chain C) were structurally aligned to the AF-O07091-F1 prediction and fused into a continuous model to reconstruct the whole cytochrome c, PscC subunit. Only PscC residues 5-109 were extracted from pdb 7z6q chain C. The missing residues between 1-5 and 109-129 were reconstructed from the AF-O07091-F1 prediction and fused to the experimentally resolved and aligned structures. Thus, one cytochrome c, PscC chimera was built, bound to PscA (chain A) from pdb 7z6q. All other chains of the Cryo-EM structure were disregarded. PscA (Chain A) from pdb 7z6q and the PscC chimera were dimerized to build the dimeric reaction centre bound to two PscC subunits. For the dimeric structure two different models were considered; the first with two PscC (2xPscA-2xPscC) and the second with just one PscC unit bound to the dimeric PscA/PscA reaction centre (2xPscA-1xPscC). All resolved lipids and water molecules were retained in the structure. The protonation states of Glu, Asp residues in chains PscA-PscC were predicted by from the PROPKA method (Olsson et al. 2011; Søndergaard et al. 2011) PDB2PQR server at neutral pH (https://server.poissonboltzmann.org/). In detail, for PscA all His residues were protonated only at the N_δ_ sites, except His-270 and 290 that were protonated only at the N_ε_ sites. Asp-110, Asp-563 and Glu-68 were kept always protonated, whereas all other acidic residues were left deprotonated. For the chimeric PscC, His-39 and His-156 were protonated only at the N_δ_ sites and His-203 only at the Nε site. All Asp and Glu residues were treated as deprotonated for chain C. These (de) protonations are vital to produce the correct hydrogen bonding network in chains A (PscA) and C (chimeric PscC) so that no large deviations are observed from the CryoEM, or x-ray experimentally resolved structures.

The model structures were embedded in a POPC (1-palmitoyl-2-oleoyl-glycero-3-phosphocholine) membrane patch of around 570 lipids and hydrated by around 79500 TIP3P water molecules (lipid-water ratio at ~139.5) (Mark and Nilsson 2001). The models contained 150 mM KCl, with a surplus of Cl^-^ ions (13.6mM) to neutralize the system. The models contain between ~344k (2xPscA-1xPscC) and ~347k (2xPscA-2xPscC) atoms. The equilibrated unit cell dimensions of each model were roughly 15.5 x 15.5 x 14.4 nm^3^ in dimensions.

***Model Parameterization***. The Charmm36 Force Field(Lindahl et al. 2010) was employed for the polypeptide chains and heme groups. The bacteriochlorophylls pigments were parameterized based on the literature (Chandrasekaran et al. 2015), thylakoid lipids bound within the protein scaffold and POPC lipids of the membrane patch were described by Charmm compatible parameters in the harm gui (Jo et al. 2008)

***Model equilibration.*** Based on published protocols, all models were relaxed and equilibrated with gradual removal of constraints on the protein backbone-heavy atoms(Daskalakis et al. 2020). Briefly, a series of constant volume (nVT), and constant pressure (nPT) ensemble runs, increased the temperature from 100K to 319K prior to the production runs. Classical MD simulations were run for further equilibration for 1 μs. Newton’s equations of motion were integrated with a time step of 2.0 fs. The leapfrog integrator in GROMACS 2020 was employed (Berendsen et al. 1995). The runs have been performed in the constant pressure nPT ensemble with semi-isotropic couplings in the x-y membrane plane and in the z-direction (compressibility at 4.5 x 10^-5^). Moreover, the van der Waals interactions were smoothly truncated between 1.0 - 1.2 nm with the Verlet cut-off scheme (force-switch algorithm and tolerance at 10^-3^). Short-range electrostatic interactions were truncated at 1.2 nm and long-range contributions were computed within the Particle-Mesh-Ewald (PME) approximation with the tolerance at 10^-5^ and Fourier spacing at 0.15 (Darden et al. 1993). All hydrogen-heavy atom bond lengths were constrained employing the LINCS algorithm(Hess et al. 1997). The v-rescale thermostat is employed (Bussi et al. 2007) (temperature coupling constant = 0.5) and the C-rescale barostat(Bernetti and Bussi 2020) (1 atm; pressure coupling constant = 2.0).

***Production runs***. After the extended equilibration of the models by classical MD (500ns) at 319K, the replica exchange (RE) enhanced sampling method with solute tempering (REST2) (Wang et al. 2011) was employed. The whole PscC subunits were considered as the solute. Twenty replicas were considered for each RE run for 200ns each at equivalent temperatures between 319-500K. The first 50ns of all replicas were considered as further equilibration time (without exchanges) at each effective temperature. This totals in a 4μs RE simulation time. An exchange attempt was performed every 1000 steps (2 ps) with an acceptance rate of 9 - 12% between replicas. All run parameters for the RE were identical to those of the classical MD previously described (see 1 μs equilibration parameters). The median structures of the three most populous clusters of structures in the demuxed trajectory at 319K were extracted by employing the Patrick-Jarvis method in GROMACS. Two independent classical MD trajectories were initiated from these three median structures for 1μs each at 319K. The two models with two, or just one PscC subunits were considered bound to the reaction center dimer (2xPscA-1xPscC, 2xPscA-2xPscC).


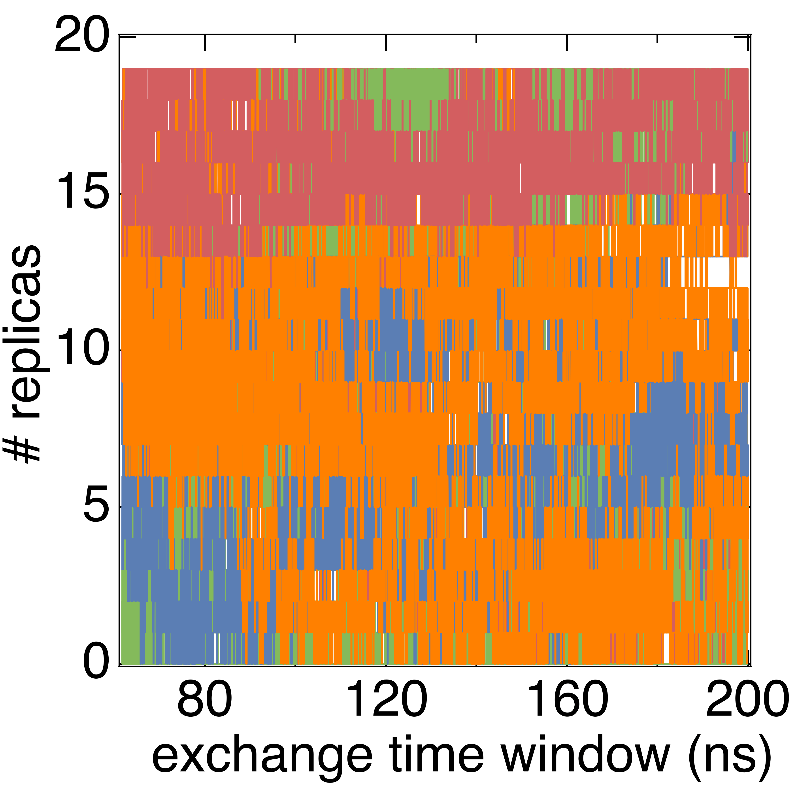


**Figure S1 |** The diffusion of replicas along different temperatures over the considered production simulation REST2 time (50-200ns). The replicas were colored in groups for visualization efficiency. Initially cool replicas (0-4) are colored in green, warmer replicas (5-9) are colored in blue, while increased-temperature replicas are consequently colored in orange (10-14) and red (15-19). The temperature range considered is between 319 K (replica 0) to 500K (replica 19).

**Graph Theory Method**. The independent classical MD production trajectories with two PscC subunits were concatenated (total simulation time of 4.8μs, **Fig. 2A**). The median structure of the most populous cluster of structures in the concatenated trajectory was extracted and converted into a graph network employing the RING method.(Clementel et al. 2022) This structure falls within the cluster where we had sampled the shortest distances between the two PscC subunits and between the docked PscC-PscA (P_840_). The residues along the shortest pathway from the distant PscC, to the docked PscC subunit and finally to the special pair P_840_ was calculated by GePhi (Bastian et al. 2009). This forms an allosteric pathway by a graph-theoretical approach (Negre et al. 2018). Perturbations at one residue can create long-range allosteric effects by their propagation through the network.

**Electron transfer pathway.** The PscA-PscC supercomplex was truncated to include any residue within 1.5nm from the residues along the allosteric pathway identified, the bacteriophylls in the special pair P840 and the two heme groups in PscC soluble domains. The electron transfer pathway between the heme groups and P840 was identified based on the method described in detail elsewhere (eMap server) (Tazhigulov et al. 2019).


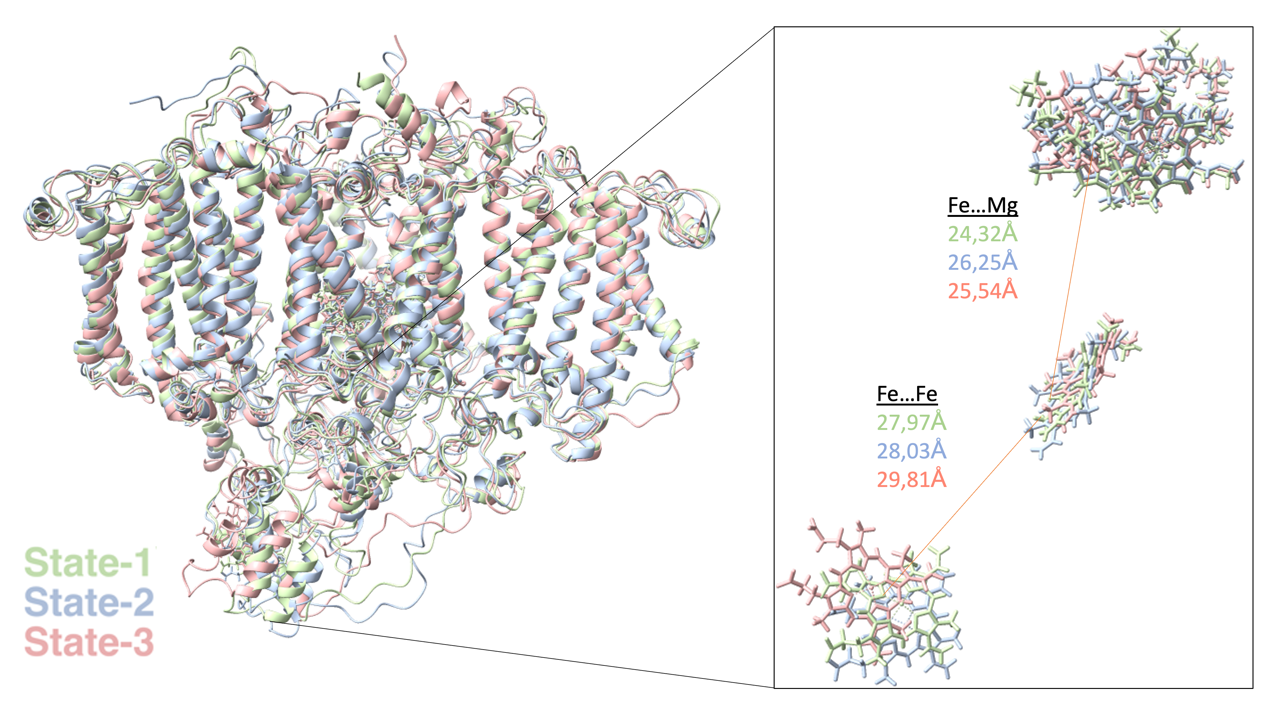


**Figure S2.** The three main conformations sampled at the REST2 method referred to as “states-1, 2 and 3” and the distances of Fe^…^Fe from PscC distant to PscC docked subunit and Fe^…^ Mg from the docked PscC subunit to the P_840_ special pair of the PscA homodimer.


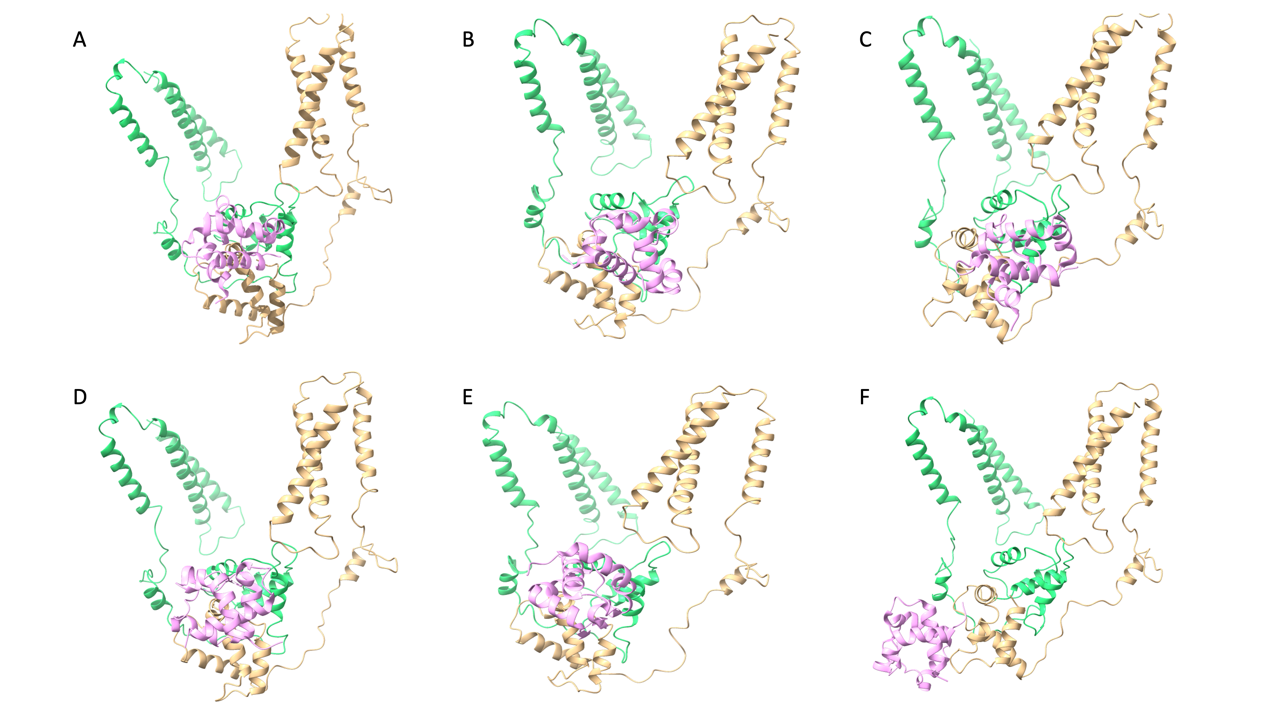


**Figure S3.** Hdock server result showing the docking of the two soluble crystal structures of CT0073 and CT0075 code always in pink. A, B and C docking of CT0073 with states-1, 2 and 3 of **Figure S2** respectively while the D, E and F the same for the CT0075  (Yan et al. 2020). The docking experiment took place using the core complex (2 x PscA and 2 x PscC and the structures of the soluble parts predicted from Alphafold2) and the crystal structures of CT0073 and CT0075 ( pdb code: 8HN3 and 4J20 respectively). To show a clear and informative figure the two PscA subunits are hidden. PscC docked (green) and PscC distant (yellow).

**
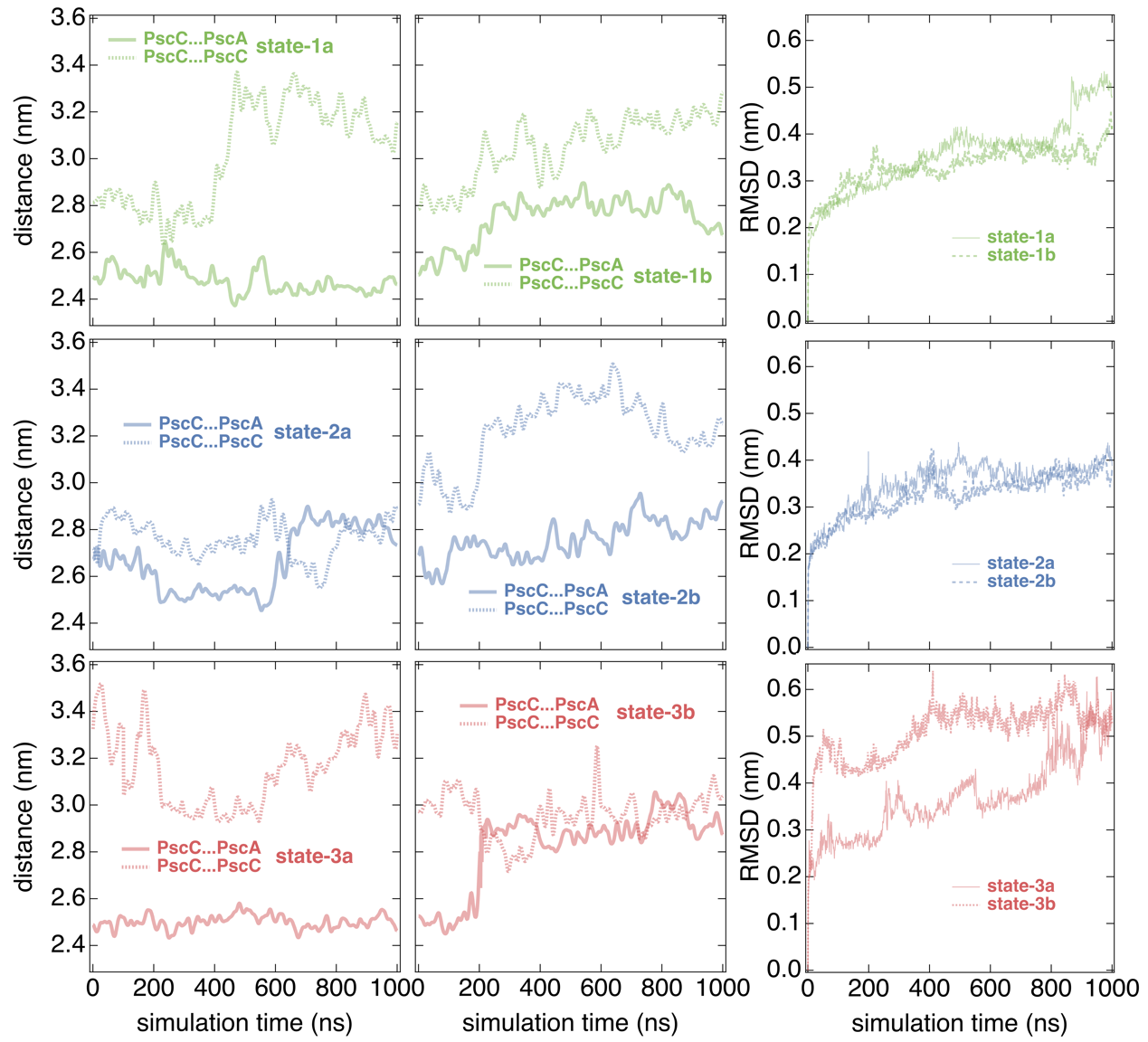
**

**Figure S4** | The dynamics sampled in the individual 6 trajectories for the Fe^…^Fe distance from PscC distant to PscC docked subunit and the Fe^…^ Mg distance from the docked PscC subunit to the P_840_ special pair of the PscA homodimer. States-1, 2 and 3 refer to the initial structures/ conformations extracted at the REST2 sampling and “a” – “b” to the two independent trajectories per initial structure (see main manuscript). Root Mean Square Deviations (RMSDs) for the whole PscA-PscCs protein backbone are also shown for the independent trajectories.

**References**

Akdel M, Pires DE V, Pardo EP, et al (2022) A structural biology community assessment of AlphaFold2 applications. Nat Struct Mol Biol 29:1056–1067. https://doi.org/10.1038/s41594-022-00849-w

Bastian M, Heymann S, Jacomy M (2009) Gephi: an open source software for exploring and manipulating networks. Icwsm 8:361–362

Berendsen HJC, van der Spoel D, van Drunen R (1995) GROMACS: A message-passing parallel molecular dynamics implementation. Comput Phys Commun 91:43–56. https://doi.org/10.1016/0010-4655(95)00042-E

Bernetti M, Bussi G (2020) Pressure control using stochastic cell rescaling. J Chem Phys 153:114107. https://doi.org/10.1063/5.0020514

Bussi G, Donadio D, Parrinello M (2007) Canonical sampling through velocity rescaling. Journal of Chemical Physics 126:. https://doi.org/10.1063/1.2408420

Chandrasekaran S, Aghtar M, Valleau S, et al (2015) Influence of Force Fields and Quantum Chemistry Approach on Spectral Densities of BChl a in Solution and in FMO Proteins. J Phys Chem B 119:9995–10004. https://doi.org/10.1021/acs.jpcb.5b03654

Clementel D, Del Conte A, Monzon AM, et al (2022) RING 3.0: fast generation of probabilistic residue interaction networks from structural ensembles. Nucleic Acids Res 50:W651–W656. https://doi.org/10.1093/nar/gkac365

Darden T, York D, Pedersen L (1993) Particle mesh Ewald: An N ⋅log( N ) method for Ewald sums in large systems. J Chem Phys 98:10089–10092. https://doi.org/10.1063/1.464397

Daskalakis V, Papadatos S, Stergiannakos T (2020) The conformational phase space of the photoprotective switch in the major light harvesting complex II. Chemical Communications 56:11215–11218. https://doi.org/10.1039/d0cc04486e

Hess B, Bekker H, Berendsen HJC, Fraaije JGEM (1997) LINCS: A Linear Constraint Solver for molecular simulations. J Comput Chem 18:1463–1472. https://doi.org/10.1002/(SICI)1096-987X(199709)18:12<1463::AID-JCC4>3.0.CO;2-H

Hirano Y, Higuchi M, Azai C, et al (2010) Crystal structure of the electron carrier domain of the reaction center cytochrome c(z) subunit from green photosynthetic bacterium Chlorobium tepidum. J Mol Biol 397:1175–1187. https://doi.org/10.1016/j.jmb.2010.02.011

Jo S, Kim T, Iyer VG, Im W (2008) CHARMM-GUI: A web-based graphical user interface for CHARMM. J Comput Chem 29:1859–1865. https://doi.org/https://doi.org/10.1002/jcc.20945

Jumper J, Evans R, Pritzel A, et al (2021) Highly accurate protein structure prediction with AlphaFold. Nature 596:583–589. https://doi.org/10.1038/s41586-021-03819-2

Lindahl E, Bjelkmar P, Larsson P, et al (2010) Implementation of the charmm force field in GROMACS: Analysis of protein stability effects from correction maps, virtual interaction sites, and water models. J Chem Theory Comput 6:459–466. https://doi.org/10.1021/ct900549r

Mark P, Nilsson L (2001) Structure and dynamics of the TIP3P, SPC, and SPC/E water models at 298 K. Journal of Physical Chemistry A 105:9954–9960. https://doi.org/10.1021/jp003020w

Negre CFA, Morzan UN, Hendrickson HP, et al (2018) Eigenvector centrality for characterization of protein allosteric pathways. Proceedings of the National Academy of Sciences 115:E12201–E12208. https://doi.org/10.1073/pnas.1810452115

Olsson MHM, SØndergaard CR, Rostkowski M, Jensen JH (2011) PROPKA3: Consistent treatment of internal and surface residues in empirical p K a predictions. J Chem Theory Comput 7:525–537. https://doi.org/10.1021/ct100578z

Søndergaard CR, Olsson MHM, Rostkowski M, Jensen JH (2011) Improved treatment of ligands and coupling effects in empirical calculation and rationalization of p K a values. J Chem Theory Comput 7:2284–2295. https://doi.org/10.1021/ct200133y

Tazhigulov RN, Gayvert JR, Wei M, Bravaya KB (2019) eMap: A Web Application for Identifying and Visualizing Electron or Hole Hopping Pathways in Proteins. J Phys Chem B 123:6946–6951. https://doi.org/10.1021/acs.jpcb.9b04816

Wang L, Friesner RA, Berne BJ (2011) Replica exchange with solute scaling: A more efficient version of replica exchange with solute tempering (REST2). Journal of Physical Chemistry B 115:9431–9438. https://doi.org/10.1021/jp204407d

Xie H, Lyratzakis A, Khera R, et al (2023) Cryo-EM structure of the whole photosynthetic reaction center apparatus from the green sulfur bacterium Chlorobaculum tepidum. Proc Natl Acad Sci U S A 120:. https://doi.org/10.1073/pnas.2216734120

Yan Y, Tao H, He J, Huang SY (2020) The HDOCK server for integrated protein-protein docking. Nat Protoc 15:1829–1852. https://doi.org/10.1038/S41596-020-0312-X
